# An Italian case series' description of thiamine responsive megaloblastic anemia syndrome: importance of early diagnosis and treatment

**DOI:** 10.1186/s13052-023-01553-1

**Published:** 2023-11-30

**Authors:** Francesca Di Candia, Valentina Di Iorio, Nadia Tinto, Riccardo Bonfanti, Claudio Iovino, Francesco Maria Rosanio, Ludovica Fedi, Fernanda Iafusco, Francesca Arrigoni, Rita Malesci, Francesca Simonelli, Andrea Rigamonti, Adriana Franzese, Enza Mozzillo

**Affiliations:** 1 Department of Translational Medical Science, Section of Pediatrics, Regional Centre of Pediatric Diabetes, Federico II University of Naples, Via S. Pansini 5, Naples, 80131 Italy; 2 Multidisciplinary Department of Medical, Surgical and Dental Sciences, Eye Clinic, University of Campania Luigi Vanvitelli, Naples, Italy; 3 Department of Molecular Medicine and Medical Biotechnology, University of Naples “Federico II”, Naples, Italy; 4 CEINGE Advanced Biotechnology, Naples, Italy; 5 Department of Pediatrics, Diabetes Research Institute, IRCCS Ospedale San Raffaele, Milan, Italy; 6 Vita Salute San Raffaele University, Milan, Italy; 7 Unit of Audiology, Department of Neurosciences, Reproductives and Odontostomatologic Sciences, University of Naples ‘’Federico II’’, Naples, Italy

**Keywords:** Nonautoimmune diabetes, Optic atrophy, Sensorineural deafness, Thiamine-responsive megaloblastic anemia syndrome, Case series

## Abstract

**Background:**

Individuals with thiamine-responsive megaloblastic anemia (TRMA) mainly manifest macrocytic anemia, sensorineural deafness, ocular complications, and nonautoimmune diabetes. Macrocytic anemia and diabetes may be responsive to high-dosage thiamine treatment, in contrast to sensorineural deafness. Little is known about the efficacy of thiamine treatment on ocular manifestations.

**Cases presentation:**

Our objective is to report data from four Italian TRMA patients: in Cases 1, 2 and 3, the diagnosis of TRMA was made at 9, 14 and 27 months. In 3 out of 4 subjects, thiamine therapy allowed both normalization of hyperglycemia, with consequent insulin suspension, and macrocytic anemia. In all Cases, thiamine therapy did not resolve the clinical manifestation of deafness. In Cases 2 and 3, follow-up showed no blindness, unlike Case 4, in which treatment was started for megaloblastic anemia at age 7 but was increased to high doses only at age 25, when the genetic diagnosis of TRMA was performed.

**Conclusions:**

Early institution of high-dose thiamine supplementation seems to prevent the development of retinal changes and optic atrophy in TRMA patients. The spectrum of clinical manifestations is broad, and it is important to describe known Cases to gain a better understanding of this rare disease.

## Background

Thiamine-Responsive Megaloblastic Anemia syndrome (TRMA, OMIM:249270), also named Rogers syndrome [1], is an autosomal recessive disease caused by loss-of-function mutations in the SLC19A2 gene (chromosome 1q23.3), which encodes the human thiamine transporter 1 (h-THTR1) protein, the main transporter of thiamine in many tissues. Disruption of this transport mechanism leads to a reduction in the intracellular concentration of thiamine, which is essential for many functions and cell survival, resulting in apoptosis and malfunction of cochlear cells, pancreatic islet cells, and haematopoietic stem cells in particular [2]. TRMA should be promptly suspected if the triad of progressive sensorineural deafness, nonautoimmune diabetes mellitus, and thiamine-responsive macrocytic anemia is present [3, 4]. However, this transporter is expressed in other tissues, and its deficiency may cause other less frequent clinical manifestations [5], such as congenital heart defects and arrhythmias [6–8], seizures and strokes [8]. In TRMA syndrome, the defect in h-THTR1 does not allow absorption of thiamine, a water-soluble vitamin B assumed at conventional doses by diet [9]. Tissues can compensate for the altered intracellular thiamine transport by using human thiamine transporter 2 (h-THTR2), encoded by the SLC19A3 gene, which has a ubiquitous distribution that is reduced/absent in the organ targets of TRMA [3]. The intake of high doses of thiamine allows the resolution of some clinical manifestations [3, 4] thanks to the activation of other low-affinity thiamine transporters. In fact, both nonautoimmune diabetes and macrocytic anemia respond to treatment with high-dose thiamine, the former to a variable extent, which can delay/prevent its onset. Instead, hearing loss appears to be progressive and irreversible and is not affected or prevented by precocious treatment, probably because cochlear cells are very sensitive to thiamine deficiency, with hearing damage perhaps manifesting as early as fetal life [10–12]. In addition, due to the rarity of the disease, the prognosis of the risk of optic nerve atrophy and retinal dystrophy, described in some patients with TRMA, is unclear. In fact, literature to date is lacking of both data relating long-term prognosis of ocular pathology in patients treated early with high-dose thiamine [12], and of data regarding which is the therapeutic dose of thiamine to administer in this rare disease.

We describe the clinical course of four Caucasian paediatric patients, known to date being the only genetically confirmed Cases in Italy. Three of them were precociously treated with high dose of thiamine, two of them (Case 2 and Case 3) early and long-term treated; of two of them (Case 3 and Case 4) we show the peculiarity of ocular manifestations.

## Case presentation

### Case 1

**Fig. 1:**
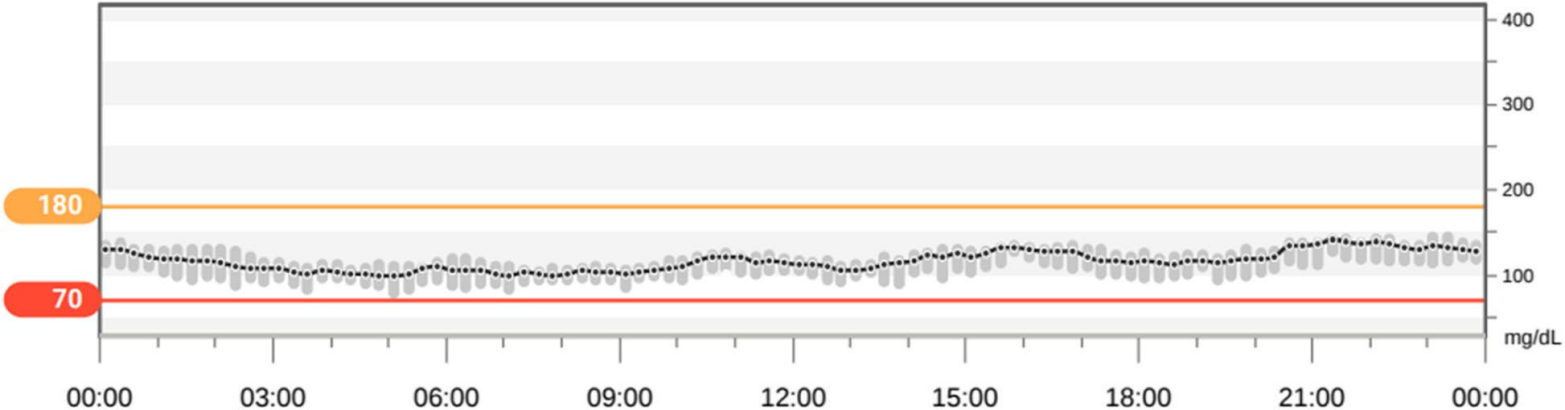
Case 1 ambulatory glucose profile report. Glycaemic control recorded by RT-CGM one week after switching from insulin therapy to oral thiamine therapy

A 9-month-old male patient, who was the only child of nonconsanguineous parents, came to the Paediatric Diabetes Centre of Federico II University in Naples for glycosuria and ketonuria, performed due to inappetence and vomiting in suspicion of a urinary tract infection. Until then, the child had shown clinical well-being and regular psychomotor development. Perinatal history: born at term by spontaneous delivery, birth weight appropriate for gestational age, not history of hyper/hypoglycaemia at birth. Family history was characterized by autoimmune thyroiditis in the maternal line. Physical examination showed mild dehydration and weight loss. Laboratory tests detected hyperglycaemia in the absence of acidaemia and led to diagnosis of nonautoimmune diabetes: autoantibodies for type 1 diabetes were negative, C-peptide 0.3 ng/ml, glycosylated hemoglobin (HbA1c) 9.7% (83 mmol/mol). Basal-bolus insulin therapy (0.22 U/kg/day) and real-time continuous glucose monitoring (rtCGM) were started. The blood count (CBC) showed age-matched hypo regenerative macrocytic anaemia: haemoglobin (Hb) 7.5 g/dl; haematocrit (HCT) 23%; mean corpuscular volume (MCV) 97 fl; reticulocyte production index 0.76%; other cell populations within limits. Peripheral blood smear was not performed. Iron, ferritin, folate and vitamin B12 levels were all within normal limits. Based on the early onset of diabetes and the absence of beta-cell autoantibodies, a genetic test for monogenic diabetes was performed by next-generation sequencing (NGS) [13]. In the suspicion of TRMA syndrome, audiometric and ophthalmology examinations were performed. The auditory brainstem response (ABR) showed bilateral sensorineural hearing loss. Orthoptic examination with inspection, pupillary reflexes, ocular motility, Hirschberg corneal reflex test, cover test, and monocular tracking were performed; all the examinations were normal. The child was dilated with tropicamide and underwent fundus examination with indirect ophthalmoscopy; the lens was clear, and no retinal changes were detected. A compound heterozygous mutation c.242dupA (p. Tyr81*) and c.1370delT (p. Leu457*) located on exons 2 and 6 of the SLC19A2 gene, respectively, was detected. The patient’s father was a carrier of c.1370delT, and the mother was c.242dupA. Treatment with oral thiamine (300 mg/day) was started until discontinuation of insulin obtained after 24 h. The glycaemic control recorded by RT-CGM one week after the completed switching from insulin therapy to oral thiamine therapy; the ambulatory glucose profile showed these metrics: mean blood glucose 113 mg/dl; time in range (TIR) 96.2%; estimated glycosylated haemoglobin 5.6% (Fig. 1). Two weeks after thiamine, anaemia resolved (Hb 12.8 g/dl; MCV 72 fl).

At 13 months, the patient underwent cochlear implantation due to sensorineural hearing loss. A few hours after the end of the surgery, because of the use of cortisone for periauricular oedema, despite a further increase in the dose with thiamine, the child showed hyperglycaemia, confirmed by continuous glucose monitoring (data not shown). Insulin therapy was restarted in addition to thiamine treatment. A few days after cortisone suspension and inflammation resolution, rapid improvement in blood glucose was observed, and insulin therapy was again discontinued, and the patient continued thiamine alone at a dose of 600 mg/day. The electrocardiogram performed before surgery to implant the prosthesis was normal. To date, the patient aged 18 months of life assumes a thiamine dose of 480 mg/day, which has been adjusted during the follow-up to normalize Hb and MCV. The patient shows sensorineural hearing loss treated with cochlear implantation, absence of both ocular involvement and diabetes mellitus, probably thanks to the precocious high dose thiamine treatment (Table 1).

**Table 1:** Clinical features of Italian children affected by TRMA

	**Case 1**	**Case 2**	**Case 3**	**Case 4**
**Consanguineous**	No	No	No	No
**Age of symptom onset (months)**	9	20	20	27
**Onset symptoms**	Hyperglycaemia Macrocytic anaemia Deafness	Hyperglycaemia Macrocytic anaemia Deafness	Hyperglycemia Deafness Macrocytic anemia Retinitis pigmentosa Optic atrophy	Hyperglycemia Deafness Macrocytic anemia Peripheral pigmentary retinal alterations
**Latency start of thiamine therapy**	2 months	Few days	7 years	Few days
**Dose of oral thiamine**	480 mg/die	300 mg/die	600 mg/die	1200 mg/die
**Insulin dependent diabetes**	No	No	Yes	No
**Ophthalmic Features**	Not present	Not present	Retinal dystrophy Optic atrophy	Not present
**Cardiac features**	Not present	Sporadic junctional rhythm with normal heart rate	Not present	Not present
**Molecular finding**	Compound heterozygous for c.242dupA (p.Tyr81*) in exon 2 and c.1370delT (p.Leu457*) in exon 6	Homozygous for c.1370delT (p.Leu457*)	Compound heterozygous for c.242dupA (p.Tyr81*) in exon 2 and c.1370delT (p.Leu457*) in exon 6	Compound heterozygous for c.242dupA (p.Tyr81*) in exon 2 and c.1370delT (p.Leu457*) in exon 6

### Case 2

A 14-month-old male patient, a second child born to a nonconsanguineous couple, came to the Department of Endocrinology at San Raffaele Paediatric Medical Centre in Milan for hyperglycaemia (281 mg/dL) during viral pneumonia due to H3N2 influenza. No family history of health issues. He was born at term from vaginal delivery with pregnancy characterized by polyhydramnios and macrosomia (birth weight 3940 g).

The patient presented fever, tachycardia, tachypnoea, fatigue, myalgia, malaise, and mild dehydration at clinical examination.

Laboratory tests detected hyperglycaemia (202 mg/dL; 11.2 mmol/L) without ketoacidosis (pH 7,39 HCO3 19,2 BEB -5,1) and HbA1c 6,2% (45 mmol/mol). Diagnosis of nonautoimmune diabetes was made due to the negativity of autoantibodies for autoimmune diabetes. Since CBC showed macrocytic anaemia (Hb 7.5 g/dL; HCT 27,9%, RBC 2450000/L; MCV 95,1 fL; RDW 23.5) with vitamin B12 and folate within the normal range, TRMA syndrome was suspected. The ABR examination showed sensorineural hearing loss; echocardiography and electrocardiogram were normal. Genetic analysis confirmed homozygous mutation in the SLC19A2 gene [c.1370delT with premature stop codon p. (Leu457Ter)]. The parents were healthy carriers (heterozygous) of this gene mutation.

Insulin therapy at a dose of 0.5 IU/kg/day was initially administered, and then oral thiamine therapy (300 mg/day) was introduced with resolution of hyperglycaemia until complete discontinuation of insulin after 8 days. Anaemia resolved after 1 month of treatment with oral thiamine. Deafness was treated with hearing aids at 4 years. To date, after a 6-year follow-up, the patient shows nonautoimmune diabetes treated with thiamine (300 mg/day) with normal glycaemic control (HbA1c 5%); deafness treated with a hearing implant; sporadic junctional rhythm shown by the last electrocardiogram with normal heart rate, holter ECG and echocardiography. Ophthalmologic evaluation, visual evoked potentials and Optical Coherence Tomography do not show ocular involvement. The Hb value and MCV of the blood count are within the normal range (Table 1).

### Cases 3 and 4

The last two Cases are two Italian sisters whose clinical history has been previously described [3] and summarized here.

The younger sister, the third child born to healthy nonconsanguineous parents, was referred to the Paediatric Diabetes Centre of Federico II University because of nonautoimmune insulin-dependent diabetes onset at the age of 27 months and firstly treated with insulin.

She had a hearing prosthesis since the age of 18 months because of sensorineural deafness. The presence of macrocytic anaemia was also noted during the hospitalization for diabetes onset. The oldest sister (Case 4), 25 years of age, had sensorineural deafness treated with prosthesis; nonautoimmune diabetes since the age of 20 months; blindness due to retinitis pigmentosa; and optic atrophy diagnosed at the age of 36 months. In her history: transfusion at the age of 7 years due to the onset of macrocytic anaemia in another hospital.

The presence of the clinical triad of nonautoimmune diabetes, deafness and macrocytic anaemia raised suspicion of TRMA syndrome in both sisters. Molecular testing detected two heterozygous mutations, c.242dupA (p. Tyr81*) and a novel c.1370delT (p. Leu457*) in each of the two siblings.

After the molecular diagnosis of macrocytic anaemia, Case 3 began treatment with oral thiamine at a dose of 100 mg/day in addition to insulin therapy, to obtain a normalization of both Hb and MCV. Insulin therapy was suspended at age of 43 months. Currently she is 15 years old and is still off insulin therapy, maintaining good glycaemic control (last HbA1c: 5.9%), dose of thiamine 1200 mg/day, titrated during the follow-up to obtain Hb and MCV values within normal ranges. She shows deafness treated with hearing tools; normal heart rate and echocardiography; and is not blind (Table 1).

Case 4, the elder sister's, continued to require insulin treatment, though increased (200 mg/day) to achieve normalization of both Hb and MCV. Currently, the oldest sister is 36 years and shows insulin-requiring diabetes; is deaf and blind; she has solved macrocytic anaemia and with normal heart rate and echocardiography.

### Long term ocular investigations of case 3 and case 4

**Fig. 2:**
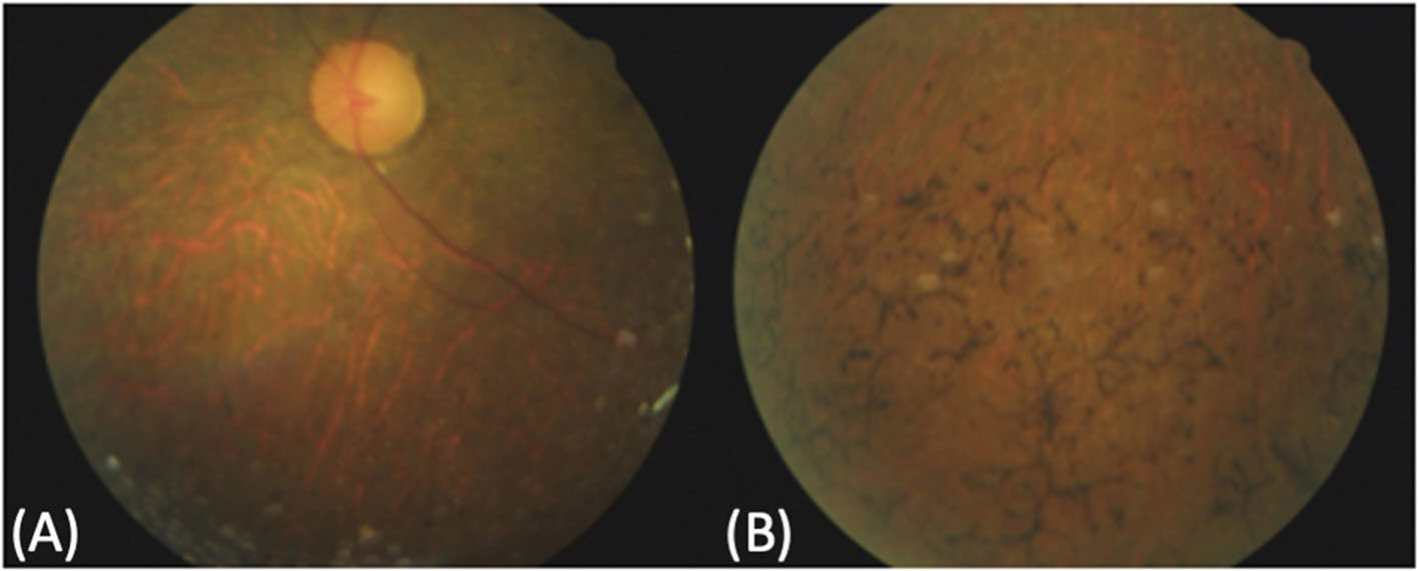
Color fundus photographs of the left eye of the older sister acquired in January 2011. Only the left was available, and the images were not centred on the posterior pole due to nystagmus. **A**: Color fundus picture of the nasal retinal sector showing optic disc pallor and attenuation of the retinal vessels. **B**: Color fundus picture of the inferior retinal sector showing bone-spicule pigment deposits in the mid periphery)

Since the time of the diagnosis of TRMA, the two sisters were referred to the Centre for Inherited Retinal Dystrophies of the Eye Clinic, University of Campania “Luigi Vanvitelli” for an ophthalmological follow-up evaluation. This centre is also currently following Case 1 which to date does not show ocular impairment. Case 3 and Case 4 underwent annual ophthalmological examinations, including best corrected visual acuity (BCVA) measurements performed using either a Snellen projection chart or Teller Acuity Cards in preschool for the little sister, slit lamp anterior segment examination and fundus examination, Goldmann visual field examination (GVF), electroretinogram (ERG) recorded according to the protocol of the International Society for Clinical Electrophysiology of Vision (ISCEV) [14], and visual evoked potentials (VEP) (LKC UTAS E3000 LKC Technologies, Inc., United States). Spectral-domain optical coherence tomography (SD-OCT, Heidelberg Engineering, Heidelberg, Germany) with near-infrared images and fundus autofluorescence (FAF) images (Spectralis HRA-OCT, Heidelberg Engineering, Heidelberg, Germany). The older sister, aged 36 years (Case 4), first presented for ophthalmologic observation at age 25 years, reporting a history of nystagmus and tapeto-retinal degeneration developed at age 3 years. At the first eye examination in January 2011, nystagmus was confirmed, and BCVA was of light perception in both eyes. Anterior segment slit lamp examination and intraocular pressure (< 20 mmHg) were normal bilaterally. Fundus examination showed a typical retinitis pigmentosa (RP) appearance with bone-spicule pigment deposits in the mid peripheral retina, optic disc pallor and attenuation of the retinal vessels (Fig. 2).

**Fig. 3:**
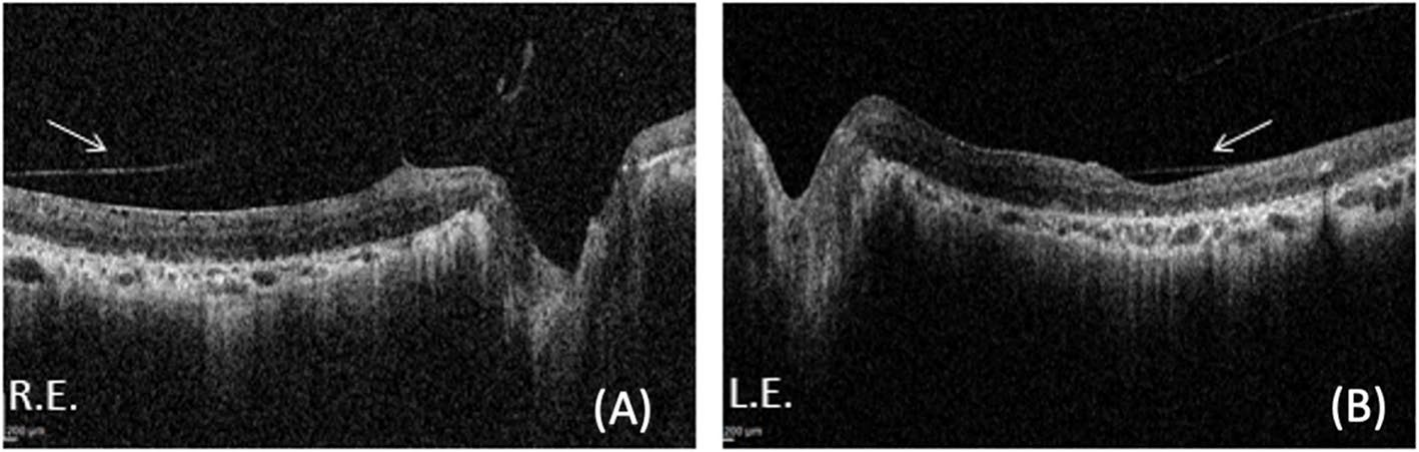
Spectral-domain optical coherence tomography (SD-OCT) of the older sister performed in September 2020. **A**: SD-OCT perifoveal B-scan of the right eye revealing vitreo-macular traction syndrome and epiretinal membrane (ERM) along with severe outer retinal layers and retinal pigment epithelium (RPE) atrophy. **B**: SD-OCT foveal B-scan of the left eye showing ERM and severe outer retinal layers and RPE atrophy)

**Fig. 4:**
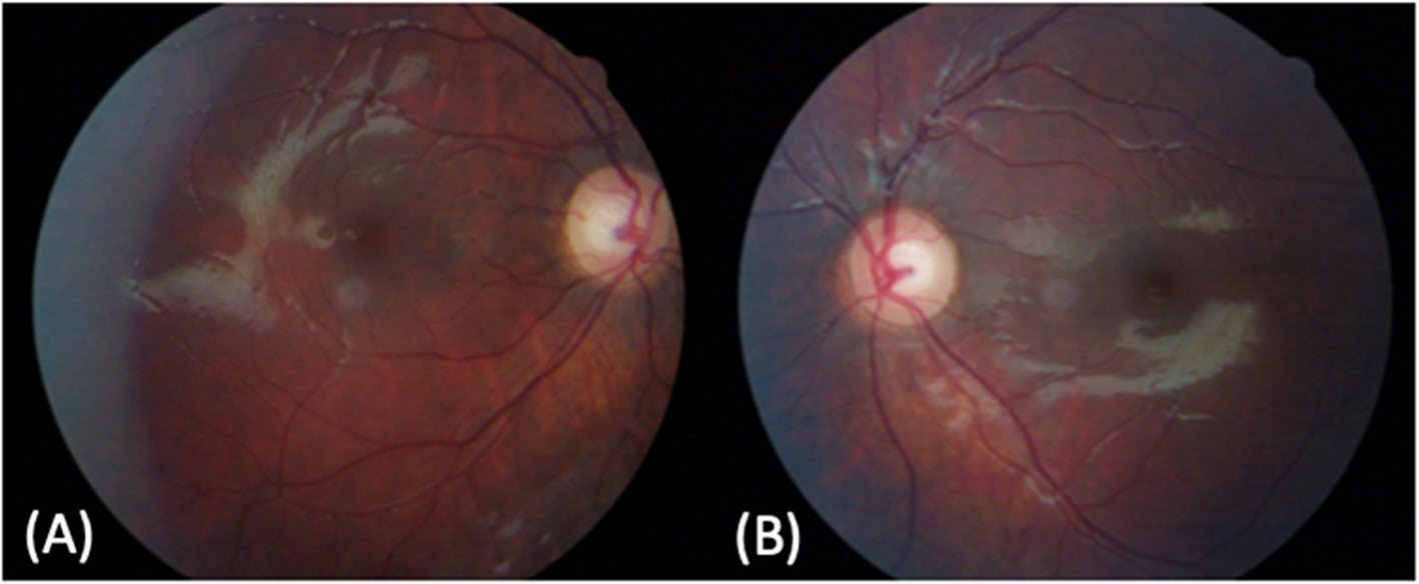
Colour fundus photographs of the right eye (RE) and left eye (LE) of the younger sister acquired in September 2020. **A** and **B**: right eye (RE) and and left eye (LE) colour fundus photographs showing mild disk pallor, with preserved retinal vessel calibre and foveal reflex

**Fig. 5:**
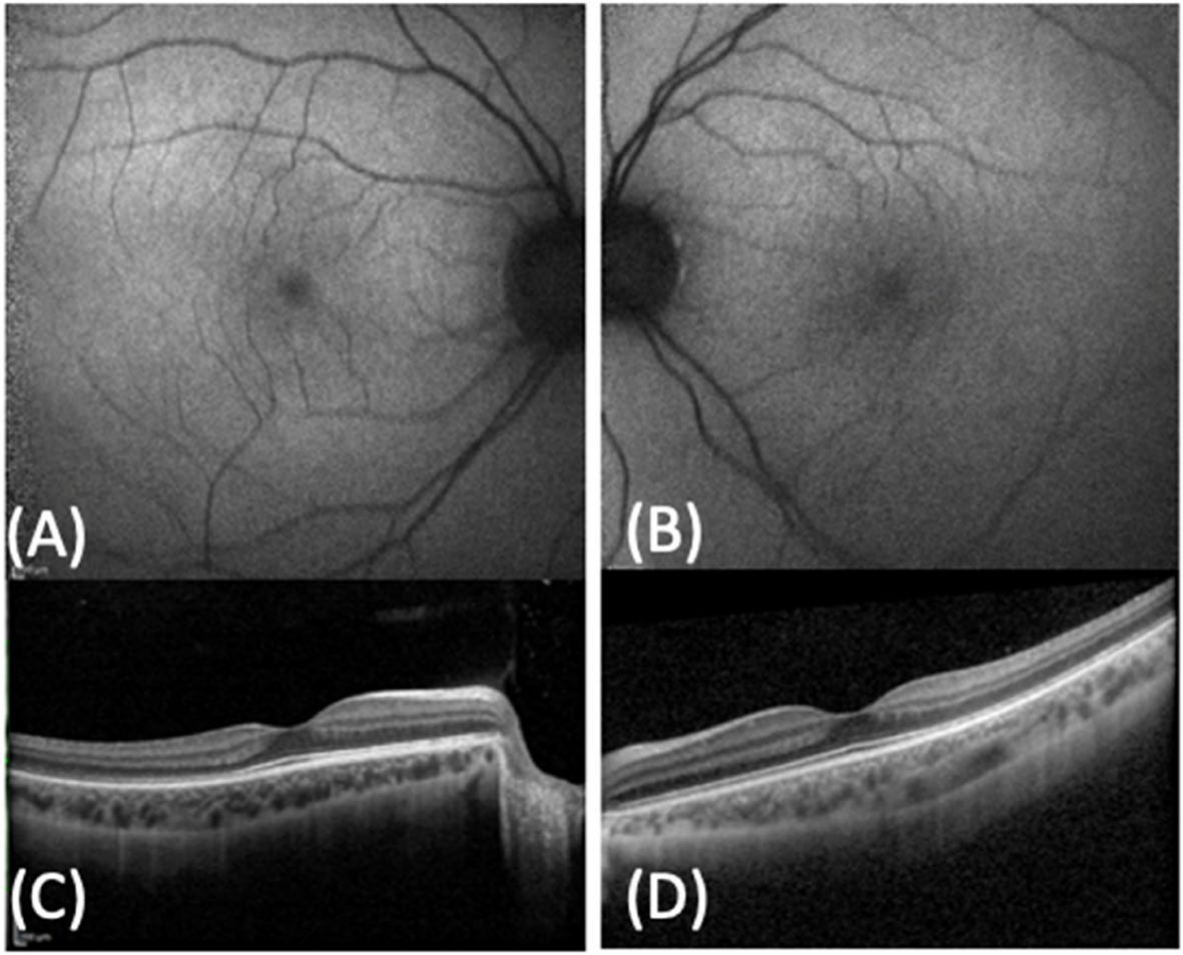
Spectral-domain optical coherence tomography, fundus autofluorescence of the younger sister acquired in September 2020. **A** and **B**: RE and LE FAF revealing a normal autofluorescence of the posterior pole. **C** and **D**: RE and LE SD-OCT showing an integrity of the inner and outer retinal layers and a normal reflectivity of the retinal pigment epithelium)

VEP responses were of increased latency and reduced amplitude, and ERG responses were nonrecordable. At the last visit, ocular involvement and responses to the electro functional tests remained unchanged. They did not detect significant changes in fundus oculi over the entire follow-up period. SD-OCT evaluation revealed the presence of a bilateral epiretinal membrane with vitreous-macular traction (VMT) in the right eye (RE) and outer retinal layer/retinal pigment epithelium (RPE) atrophy in both eyes (Fig. 3). On the first ophthalmic examination of the youngest sister at 30 months of age (Case 3), she presented no signs of nystagmus or strabismus, and her fundus appearance was normal with a bilateral mild optic disk temporal pallor. VEP responses were normal in latency and amplitude, and ERG responses were recordable.In the last follow-up in January 2022, BCVA was 20/20 in both eyes, the Ishihara colour vision test was normal, and fundus examination was stable over time, showing a normal macula and mid peripheral retina (Fig. 4) FAF revealed normal autofluorescence of the posterior pole (Fig. 5 A and B) and SD-OCT showed normal macular thickness with preservation of the inner and outer retinal layers (Fig. 5 C and D). ERG highlighted undetectable scotopic and markedly abnormal photopic responses, and CVG demonstrated progressive concentric constriction.

## Discussion and conclusions

The availability of access to genetic testing and the development of advanced DNA sequencing methods, such as next-generation sequencing (NGS), have improved the knowledge about TRMA. In fact, in recent years, numerous cases have been reported in the literature, even if TRMA is an extremely rare syndrome, and to date, its prevalence and incidence are still unknown [15] A recent literature review conducted by Zhang et al. showed 183 patients with TRMA belonging to as many families from around the world [16]. It is known that megaloblastic anaemia in TRMA may be reversed in affected patients by supraphysiological dosing of thiamine to take advantage of low affinity/high capacity transporters [17], which could solve diabetes [3], as in three described patients (Case 1, Case 2, Case 3). According to some reports, despite the initial benefit, some patients became insulin dependent after a period of insulin suspension [18, 19]. These are cases described in an era in which the molecular diagnosis of TRMA was not often performed, such cases in fact were frequently confused for Wolfram syndrome and treatment with high dose of thiamine was not executed [4]. It is possible that an early diagnosis may be essential to prevent/slow down the course of a rare disease like TRMA, particularly for ocular degenerations and glucose metabolism derangements [20]. In our cases series' description, the older sister (Case 4), though she assumed thiamine supplementation regularly (not at high dosage) after clinical diagnosis, had already developed advanced RP with severe visual impairment before molecular diagnosis, and it was never possible to stop insulin as the diabetes never resolved, unlike the other three cases described (two of these with a six- and twelve-year follow-up). Nonetheless, in some stressful situations (i.e.: surgery, inflammation, etc.) thiamine therapy may not be sufficient to control blood sugar levels (Case 1). Therefore, the precocious diagnosis of TRMA remains a challenge for the paediatric diabetologist, since a differential diagnosis is necessary with respect to a rare disease similar to Wolfram syndrome [21], of which there are two variants with clinical factors similar to TRMA [21, 22]; in fact, Case 3 was first assumed to be a patient affected by Wolfram syndrome. Thiamine therapy does not seem to modify the course of sensorineural hearing loss, which is progressive and begins in the very early stages of life [12], thus indicating an increased susceptibility to damage of the cochlear and acoustic nerve cells. The irreversibility of sensorineural hearing loss may be due to the increased demand for thiamine in cochlear and acoustic nerve cells compared to other tissues [23]. It has been hypothesized that the SLC19A2 mutation may impair the action potential in excitatory cardiac tissue [6]. In our report, patient 2 presented at the age of 6 years and 6 months with a sporadic junctional heart rhythm with normal heart rate without compromising cardiac function but probably requiring close cardiological follow-up. Limits of our cases series' description are the lack of data regarding ocular manifestations of Case 3. It was not possible to recover the specificity of the ocular test investigations, however, we collected the reports of the various eye examinations performed every year of the follow up in Milan Pediatric Diabetes Centre. Another limit, Case 2, Case 3 and Case 4 did not wear a glucose monitoring sensor while attempting to switch from insulin to high-dose thiamine. Strengths of our case series are the long-term follow-up of retinal manifestations of TRMA patients, which demonstrates the effectiveness of early administration of high-dose thiamine in preventing ocular damage and visual loss in patients with SLC19A2 gene mutations. Our Case 3 represents the longest-term follow-up described in the literature to date of a patient affected by TRMA precociously treated with high dosage of thiamine, that to date is not blind, differently by her sister. It is conceivable that early initiation of high dosage of thiamine therapy in this rare disease could prevent or improve ocular abnormalities. In addition, interestingly, all our patients share the same mutation, L457*, and even though not related, they originate from the Campania area, suggesting a clustering of the mutation in this geographic area [24]. This mutation has been classified as “pathogenic” in accordance with the American College of Medical Genetics and Genomics (ACMG) classification criteria [25] and is reported in the Human Gene Mutation Database [26] (http://www.hgmd.cf.ac.uk.ac). Obviously, the cases described are very rare; therefore, further long-term investigations, in which thiamine may be administered as early as during pregnancy in prenatally diagnosed cases, are needed to better understand whether thiamine is able to prevent or minimize the onset of some clinical manifestations, such as auditory manifestations, that do not seem to respond to oral thiamine therapy, even when started early after birth and at high dosages.

## Data Availability

The datasets generated during and/or analysed during the current study are available from the corresponding author on reasonable request.

## Abbreviations

TMRA: Thiamine-responsive megaloblastic anaemia
HbA1c: Glycosylated haemoglobin
rtCGM: Real-time continuous glucose monitoring
NGS: Next-generation sequencing
ABR: Auditory brainstem response
BCVA: Best corrected visual acuity
GVF: Goldmann visual field
ERG: Examination electroretinogram
ISCEV: International Society for Clinical Electrophysiology of Vision
VEP: Visual evoked potentials
SD-OCT: Spectral-domain optical coherence tomography
FAF: Near-infrared images and fundus autofluorescence images
VMT: Vitreous-macular traction
RPE: Retinal layer/retinal pigment epithelium atrophy
ACMG: American College of Medical Genetics and Genomics
